# The Role of TLR4 on PGC-1*α*-Mediated Oxidative Stress in Tubular Cell in Diabetic Kidney Disease

**DOI:** 10.1155/2018/6296802

**Published:** 2018-05-16

**Authors:** Shuguang Yuan, Xuemei Liu, Xuejing Zhu, Zhong Qu, Zailiang Gong, Jun Li, Li Xiao, Yuan Yang, Hong Liu, Lin Sun, Fuyou Liu

**Affiliations:** ^1^Department of Nephrology, The Second Xiangya Hospital, Central South University, Changsha, Hunan, China; ^2^Changsha Central Hospital, Changsha, Hunan, China

## Abstract

The role and precise mechanism of TLR4 in mitochondria-related oxidative damage and apoptosis of renal tubules in diabetic kidney disease (DKD) remain unclear. We examined the expression of TLR4 in renal biopsy tissues. Db/db diabetic mice and HK-2 cells cultured under high glucose (HG) were used as in vivo and vitro models. Real-time RT-PCR, Western blot, and immunohistochemistry were performed to examine the mRNA and protein levels of TLR4, NF-*κ*Β, PGC-1*α*, cytochrome C, and cleaved caspase-3. ATP level, activity of electron transport chain complex III, and antioxidant enzymes were investigated for mitochondrial function. Electron microscopy (EM) and MitoTracker Red CMXRos were used for mitochondrial morphology alteration. DHE staining and TUNEL assay were detected for ROS accumulation and apoptosis. PGC-1*α* plasmids were used for the overexpression of PGC-1*α* in HK-2. TAK242 and parthenolide were used as TLR4 and NF-*κ*B blockers, respectively. Results showed that TLR4 was extensively expressed in the renal tubules of DKD patients and db/db diabetic mice, which was positively related to the tubular interstitial damage score and urinary *β*-NAG levels. In diabetic mice, inhibition of TLR4 could reverse the decreased expression of PGC-1*α*, increased expression of cytochrome C and cleaved caspase-3, mitochondrial dysfunction and deformation, increased accumulation of ROS, and activation of tubular cell apoptosis. In vitro, inhibition of TLR4 or NF-*κ*B showed consistent results. PGC-1*α* overexpression could reverse the mitochondrial dysfunction, increased cleaved caspase-3, and apoptosis in HK-2 cells treated with HG. Data indicated that the TLR4/NF-*κ*B signaling pathway might be the upstream pathway of PGC-1*α* and promote the tubular damage of DKD by modulating the mitochondria-related oxidative damage and apoptosis.

## 1. Introduction

Toll-like receptors (TLRs) are pattern recognition receptors and play a fundamental role in the activation of innate and adaptive immune responses [1, 2]. Among the 11 human TLRs, TLR4 has been implicated in the pathogenesis of acute and chronic renal disorders such as acute kidney injury (AKI), renal fibrosis, and DKD [3, 4]. Further researches have reported that TLR4 knockout diabetic mice have reduced the expression of MyD88 and TRIF and decreased NF-*κ*B activity and the release of inflammatory cytokines and renal fibrosis [5, 6]. In addition, HG was proved to induce the overexpression of TLR4 through NF-*κ*B-dependent signaling and lead to the accumulation of ROS in podocytes [7], indicating the important role of TLR4 NF-*κ*B signaling in the mechanism of DKD progression. According to our previous study, renal tubular oxidative stress injury and apoptosis play a key role in the progression of DKD in high glucose (HG) conditions [8]. These let us speculate that activation of the TLR4-NF-*κ*B signaling pathway might be involved in mitochondrial dysfunction and mitochondria-related oxidative damage of renal tubular epithelial cell (RTEC) in hyperglycemia, which is gradually to have an extremely important effect in the progression of DKD.

The peroxisome proliferator-activated receptor *γ* coactivator-1 (PGC-1) including PGC-1*α*, PGC-1*β*, and PRC is attributed to the nuclear transcription activating factor and has intimate relationship with substance metabolism [9]. PGC-1*α* is proved to stimulate mitochondrial biogenesis and respiration through the induction of uncoupling protein 2 (UCP-2) and the regulation of nuclear respiratory factors (NRFs) [10]. In addition, our previous study has also confirmed that, by adjusting transcription factors such as NRFs, PGC-1*α* could protect mitochondrial respiratory chain function and antioxidant enzymes, so as to maintain the stability of the mitochondrial structure and function [8]. Moreover, in cardiac cells, researchers found that NF-*κ*B p65 represses PGC-1*α* activity leading to metabolic dysregulation that underlies heart dysfunction and failure [11]. However, the protective effect of PGC-1*α* on mitochondria and its relationship with TLR4/NF-*κ*B signaling path in DKD are not fully clear and need to be further investigated.

Our aim of this study is to explore the function of the TLR4/NF-*κ*B pathway in mitochondria-related oxidative damage and apoptosis of RTEC in hyperglycemia and to investigate the role of PGC-1*α* in the TLR4/NF-*κ*B pathway in DKD.

## 2. Results

### 2.1. TLR4 Expression Was Upregulated in Renal Biopsy Specimens of DKD Patients

**Figure 1:**
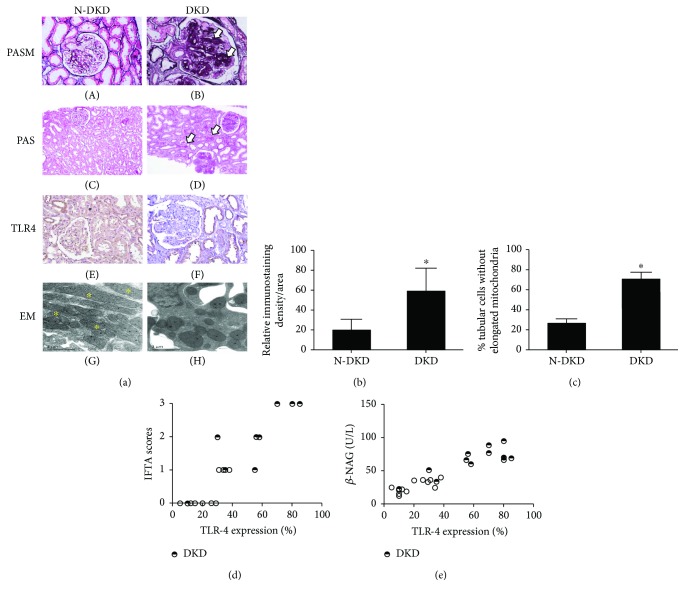
TLR4 expression was upregulated in renal biopsy specimens of DKD patients. (a) PASM (magnification ×200) and PAS (magnification ×100) staining were done to renal biopsy tissues of patients with DKD (A1, B, D) and N-DKD (A, C). IHC studies demonstrated the expression of TLR4 in renal biopsy tissues of patients with DKD versus N-DKD (F versus E). EM detected tubular mitochondria of renal tissue of DKD patients compared with N-DKD (H–I versus G) (scale bars: 1 *μ*m). Asterisks indicate elongated (>2 *μ*m) mitochondria. (b) Renal cortical relative expression of TLR4 in renal biopsies of patients with DKD and N-DKD. (c) Quantification of mitochondrial fragmentation of renal tubular cells. (d) and (e) The correlation between TLR4 expression and tubular atrophy and interstitial fibrosis (IFTA) scores (*r* = 0.76, *P* < 0.01) and urinary *β*-NAG levels (*r* = 0.89, *P* < 0.01) were observed in the scatter plots. Values are means ± SEM. ^∗^*P* < 0.05.

The clinical characteristics of the DKD patients and N-DKD as controls in this study are shown in Table 1. PASM and PAS staining showed morphological changes in both glomerular and tubulointerstitial areas, including mesangial area expansion (Figure 1(a), B, arrow), focal tubular atrophy, and interstitial fibrosis (Figure 1(a), D, arrow) in DKD patients, compared with those in non-DKD patients. Significantly, in the N-DKD group, mitochondria with an elongated cylindrical shape with organized cristae were shown by electron microscopy (Figure 1(a), G). However, diffused fragmented mitochondria were observed in DKD patients (Figure 1(a), H). The mitochondrial changes in EM were quantified (Figure 1(c)): mitochondrial length was measured in tubular cells to determine the percentage of cells that showed filamentous mitochondria less than 1% long (>2 *μ*m). ^∗^*P* < 0.05 compared with the N-DKD group. An observably enhanced TLR4 expression was demonstrated by IHC staining in the renal tubules of DKD patients (Figures 1(a), F, and 1(b)). Correlation analysis showed that TLR4 expression was positively correlated with the interstitial fibrosis and tubular atrophy (IFTA) scores and urinary *β*-NAG level as a tubular injury marker (Figures 1(d) and 1(e)).

### 2.2. Inhibition of TLR4 Protects Tubular Cell by Regulating Mitochondria-Related Proteins in Diabetic dbdb Mice

The levels of blood urea nitrogen (BUN), serum creatinine (Cr), urine protein (Upro), and urinary albumin : creatinine ratio (ACR) were significantly increased in the db/db group; ^∗^*P* < 0.05 compared with the db/m group. However, BUN, Cr, and Upro were significantly attenuated following treatment with TAK242 (Table 2), ^∗∗^*P* < 0.05 compared with the db/db mice group. These results suggested that TAK242 administration could preserve the renal function of db/db mice to a certain extent.

**Figure 2:**
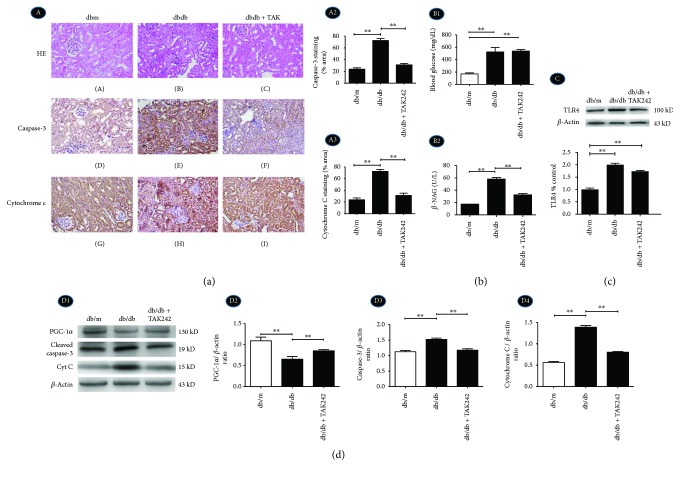
Inhibition of TLR4 protects tubular cell injury by regulating mitochondria-related proteins in diabetic dbdb mice. Dbdb mice received an intraperitoneal injection with the vehicle alone (dbdb group) or TLR4 inhibitor TAK242 (dbdb + TAK group). dbm mice were served as a control. (a) A1, A–C: HE staining was done to renal biopsy tissues of the mice (magnification ×200); A1, D–F: IHC for caspase-3 in tubular cells; A1, G–I: IHC for cytochrome C (magnification ×200); A2 and A3: semiquantification of IHC staining of caspase-3 and cytochrome C. (b) B1: Serum blood glucose level; B2: urinary excretion *β*-NAG levels in different groups. (c) Western blot for the protein expression of TLR4. (d) D1–D4: Western blot for the protein expressions of PGC-1*α*, caspase-3, and cytochrome C. Data were expressed as means ± SEM; ^∗∗^*P* < 0.01.

Loss of brush border and early tubular atrophy were observed compared with the control group by HE staining (Figure 2(a), A–C), which were ameliorated by the injection of TLR4 inhibitor TAK242. The urinary excretion of *β*-NAG, which is a marker of tubular damage, was reflected by a significant increase in diabetic dbdb mice, while it was substantially reduced by intrarenal injection of TAK242 (Figure 2(b), B2).

TLR4 was increased in dbdb mice by Western blot (Figure 2(c)). Immunohistochemistry and Western blot show a notable increase in protein expression of cytochrome C (Figures 2(a), A1, G and H, A3, and 2(d), D1, D4) and cleaved caspase-3 (Figures 2(a), A1, D–F, A2, and 2(d), D1, D3) and a decrease in PGC-1*α* (Figure 2(d), D1, D2). Their changes were markedly reversed following the injection of TAK242.

### 2.3. Inhibition of TLR4 Protects Tubular Cell from Mitochondrial-Dependent Apoptosis by Regulating Mitochondrial Structure and Function in Diabetic dbdb Mice

**Figure 3:**
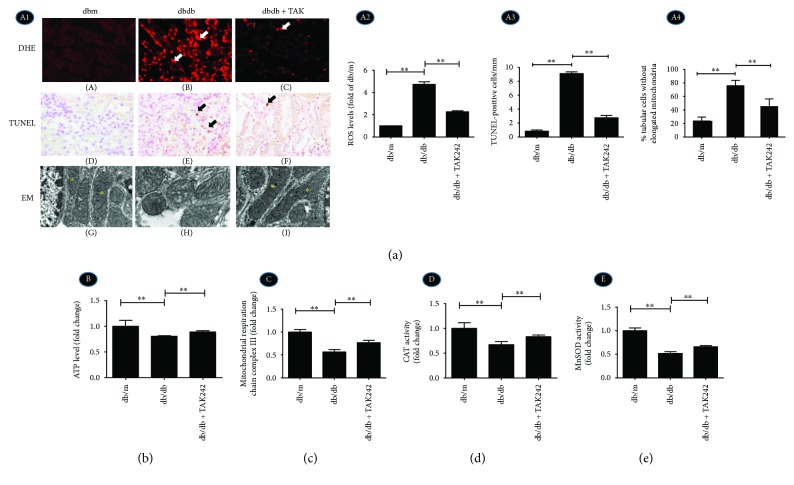
Inhibition of TLR4 protects tubular cells from mitochondrial-dependent apoptosis by regulating mitochondrial structure and function in diabetic dbdb mice. (a) A1, A–C: DHE with red fluorescence for ROS accumulation; A1, D–F: IHC of TUNEL assay for apoptosis (arrow show) (magnification ×400); A1, G–I: EM shows tubular mitochondria of renal tubular cells of dbdb mice (magnification ×20000). A2: Quantification of DHE staining expressed as fold of dbm mice. A3: Quantification of TUNEL-positive cells. A4: Relative percentage of renal tubular cells without elongated mitochondria. (b) The level of ATP production. (c) The activity of mitochondrial respiratory chain complex III. (d) Activity of CAT. (e) Activity of MnSOD. ^∗∗^*P* < 0.01. The values in (b–e) were displayed as fold change compared to the control.

ROS production was stained with red fluorescence by ROS-sensitive vital dye DHE and increased notably in the tubules of diabetic dbdb mice. Under the inhibition of TLR4 expression, ROS generation was significantly reduced (Figure 3(a), A1, A–C, A2). In addition, the inhibition of TLR4 expression dramatically reduced the degree of apoptosis in the tubular cells of diabetic dbdb mice by TUNEL assay (Figure 3(a), A1, D–F, A3). Tubular cells show elongated mitochondria with organized cristae in dbm mice (Figure 3(a), A1, G) (marked by asterisks); however, in the dbdb group, most mitochondria exhibited spherical shapes and had cristolysis (Figure 3(a), A1, H), which was partly attenuated following treatment with TAK242 (Figure 3(a), A1, I, A4).

The level of ATP production (Figure 3(b)), activity of electron transport chain complex III (Figure 3(c)), and activity of antioxidant enzymes: catalase (CAT) and manganese superoxide dismutase (MnSOD) (Figures 3(d) and 3(e)) were significantly decreased in the dbdb mouse group, which could be reversed following the injection of TAK242. These indicated that inhibition of TLR4 could attenuate the depression of mitochondrial functions in diabetic mice.

### 2.4. HG Increased TLR4 Expression and Activated NF-*κ*B p65 Phosphorylation in HK-2 Cells under HG Ambience

**Figure 4:**
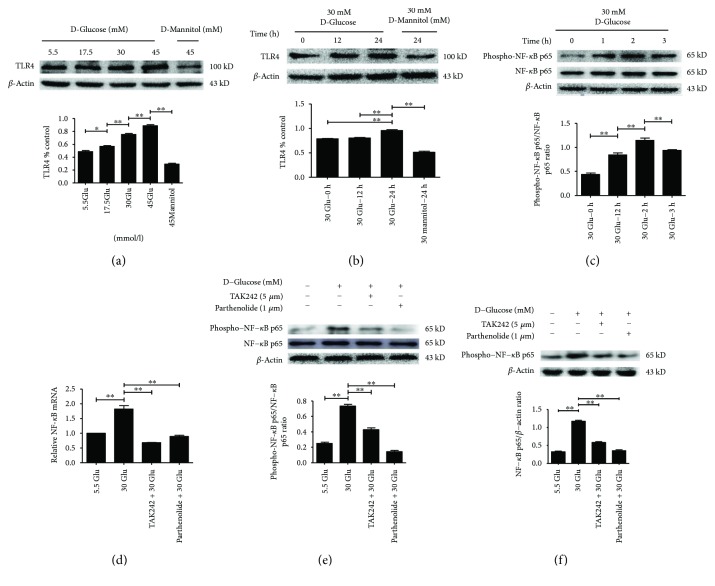
HG increased TLR4 expression and activated NF-*κ*B p65 phosphorylation in HK-2 cells. a–b: Protein levels of TLR4 in HK-2 cells following exposure to D-glucose (5.5 mM, 17.5 mM, 30 mM, and 45 mM) for 24 h (a) or D-glucose (30 mmol/L) for various times (0 h, 12 h, and 24 h) (b), and expression of NF-*κ*B p65 and phospho-NF-*κ*B p65 of cells exposed to 30 mM HG for indicated time points (0 h, 1 h, 2 h, and 3 h) (c) were determined by Western blot analysis. d–f: HK-2 cells were treated with TLR4 inhibitor (TAK242, 5 *μ*M) for 2 h prior to HG (30 mM) treatment for 2 h (e) or 24 h (f) and with NF-*κ*B blocker (parthenolide, 10 *μ*M) and HG (30 mM) for 2 h (e) or 24 h (f), and the samples were collected for RT-PCR (d) and Western blot analysis (e–f). Each assay was representative of three independent experiments. Data were expressed as means ± SEM; ^∗^*P* < 0.05 and ^∗∗^*P* < 0.01.

As shown in Figure 4, the protein level of TLR4 increased in a concentration- and time-dependent manner in HK-2 cells, and beta-actin served as a loading control (Figures 4(a) and 4(b)). In addition, the expression of phospho-NF-*κ*B p65 increased significantly in HK-2 cells treated with 30 mM HG for 2 h compared to the control (Figure 4(c)), while TLR4 inhibitor (TAK242, 5 *μ*M) [12] reversed HG-induced NF-*κ*B p65 phosphorylation (Figure 4(e)) for 2 h and for 24 h (Figure 4(f)), which indicates that NF-*κ*B is the downstream signal molecule of TLR4. Consistent with the protein expression, the mRNA level of NF-*κ*B p65 increased in HK-2 cells treated with 30 mM HG for 2 h compared to the control, while TAK242 reversed HG-induced activation of NF-*κ*B p65 phosphorylation (Figure 4(d)), which indicates that HG activated the TLR4/NF-*κ*B signaling pathway.

### 2.5. Inhibition of TLR4/NF-*κ*B Signaling Reversed the Expression of PGC-1*α* and Caspase-3 and ROS Production in HK-2 Cells under HG Ambience

**Figure 5:**
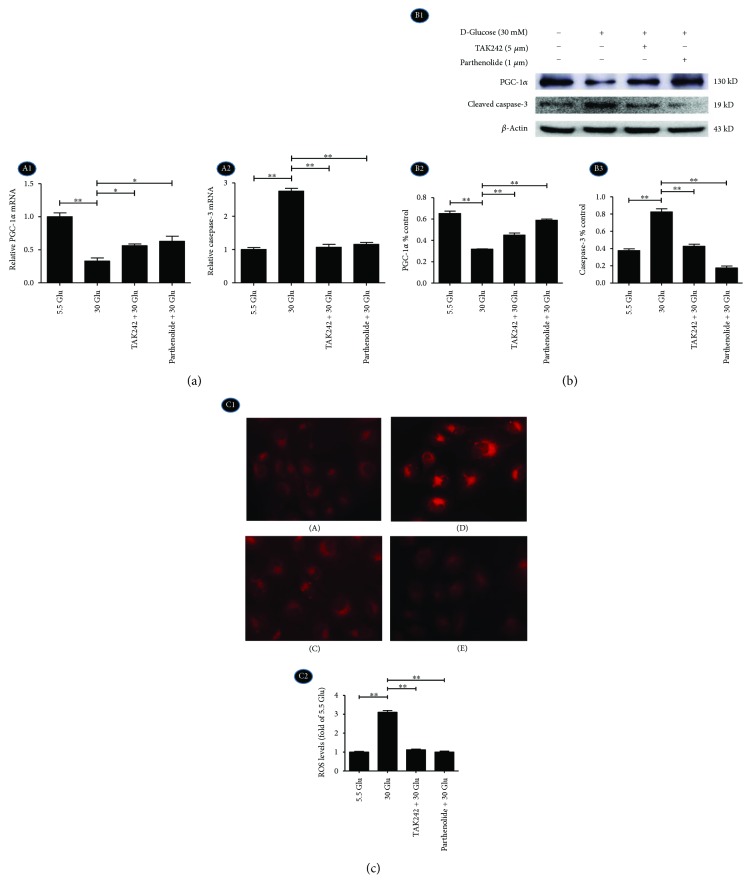
Inhibition of TLR4/NF-*κ*B signaling reversed the expression of PGC-1*α* and caspase-3 and accumulation of ROS in HK-2 cells in HG ambience. HK-2 cells were treated with TAK242 for 2 h prior to HG (30 mM) treatment for 24 h and with parthenolide and HG (30 mM) for 24 h, and samples were collected for RT-PCR (a) and Western blot analysis (b). (c) C1: MitoSOX Red staining for ROS production (magnification ×200). A: 5.5 Glu group, B: 30 Glu group, C: TAK242 + 30 Glu group, and D: parthenolide + 30 Glu group. C2: Quantification of MitoSOX Red staining. The values were expressed as fold change compared to control. Data were expressed as means ± SEM; ^∗^*P* < 0.05 and ^∗∗^*P* < 0.01.

RT-PCR (Figure 5(a), A1, A2) and Western blot (Figure 5(b), B1–B3) results showed that mRNA and protein levels of apoptosis-related protein cleaved caspase-3 were increased in the HG group (Figures 5(a), A2, and 5(b), B3), while mRNA and protein expression of mitochondria-related protein PGC-1*α* decreased in the HG group (Figures 5(a), A1, and 5(b), B2). This trend was overturned by TAK242 and NF-*κ*B blocker (parthenolide, 10 *μ*M).

For measurements of ROS, MitoSOX Red reagent (red color) was used. The cells have very low MitoSOX fluorescence in the cytoplasm of cells in the 5.5 Glu group (Figure 5(c), C1, A). HG significantly triggers an increase in mitochondrial superoxide formation (Figure 5(c), C1, B, C2). TLR4 and NF-*κ*B blocker could prevent HG-induced ROS production (^∗∗^*P* < 0.01).

These data suggested that the TLR4/NF-*κ*B pathway might be involved in HG-induced mitochondria-related ROS accumulation and apoptosis. In addition, the expression of PGC-1*α* increased in TLR4/NF-*κ*B blocked groups suggesting that PGC-1*α* might be a downstream protein of the TLR4/NF-KB signaling pathway in HG-induced changes in HK-2 cells.

### 2.6. Inhibition of TLR4/NF-*κ*B Signaling Reversed Mitochondrial Cytochrome C Release, Mitochondrial Morphology, Function, and Early Apoptosis in HK-2 Cells under HG Ambience

**Figure 6:**
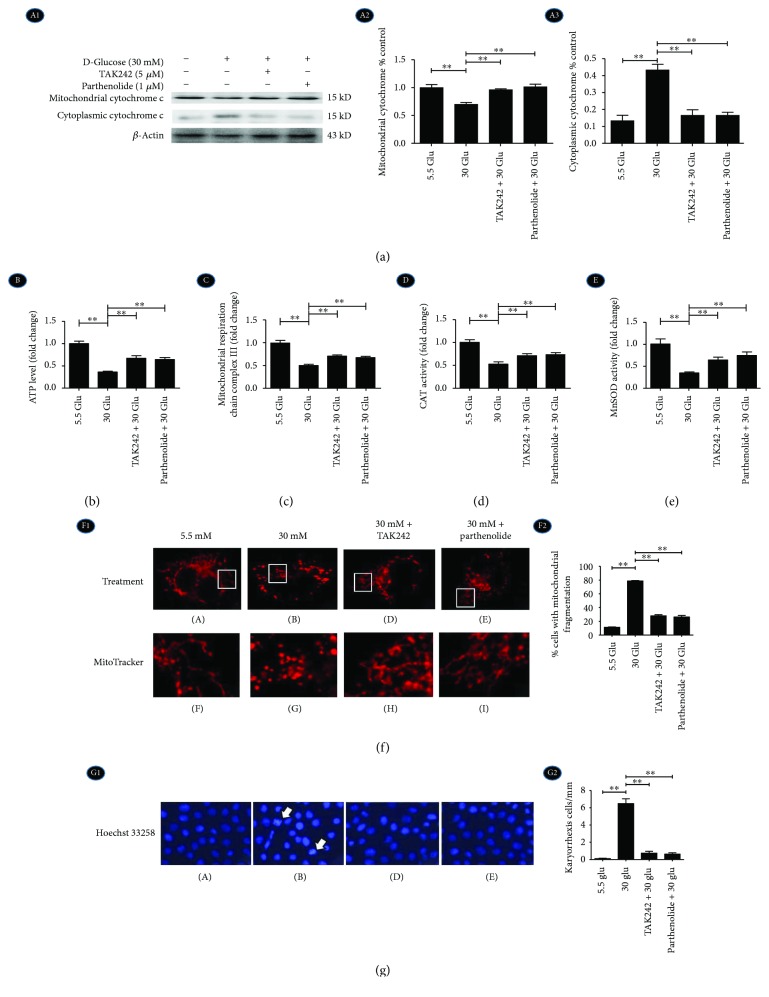
Inhibition of TLR4/NF-*κ*B signaling reversed mitochondrial cytochrome C release, mitochondrial morphology, function, and early apoptosis in HK-2 cells under HG ambience. HK-2 cells were treated with TAK242 for 2 h prior to HG (30 mM) treatment for 24 h and with parthenolide and HG (30 mM) for 24 h. (a) A1–A3: Western blot shows protein expressions of mitochondrial cytochrome C and cytoplasmic cytochrome C. (b) The level of ATP production. (c) The activity of mitochondrial respiratory chain complex III. (d) Activity of CAT. (e) Activity of MnSOD. ^∗∗^*P* < 0.01. The values in b–e were displayed as fold change compared to the control. (f) Fluorescence microscopy shows mitochondria stained with MitoTracker Red CMXRos (red) (F1) and was evaluated to determine the percentage of cells that fragmented mitochondria (F2). (g) Hoechst 33258 staining shows early apoptosis of tubular cell nuclei (blue).

At a 30 mM concentration of D-glucose, release of cytochrome C from mitochondria to cytoplasm was increased in HK-2 cells by Western blot (Figure 6(a), A1–A3), suggesting a remarkable HG-induced mitochondrial malfunction. With the intervention of the TLR4 inhibitor (TAK242) and NF-*κ*B blocker (parthenolide), the translocation of cytochrome C was ameliorated (Figure 6(a), A1–A3). The level of ATP production (Figure 6(b)), activity of electron transport chain complex III (Figure 6(c)), and activity of CAT and MnSOD (Figures 6(d) and 6(e)) were significantly decreased in the HG group, which could be reversed in the TAK242 + 30 Glu group and parthenolide + 30 Glu group. These indicated that HG activated TLR4/NF-*κ*B signaling to influence mitochondrial function.

As shown in Figure 6(f), mitochondria, which were stained by MitoTracker with red fluorescence, were filamentous with a thread-like appearance and were often interconnected to form a network in the control group (Figure 6(f), F1, A, F). The mitochondria were fragmented into spheres during HG treatment (Figure 6(f), F1, B, G), while TAK242 and parthenolide treatment reversed this trend (Figure 6, F1, D, E, H, I, F2).

In addition, Hoechst 33258 staining shows that HG (30 mM) increased karyorrhexis, which was a hint of early apoptosis, while TAK242 and parthenolide treatment reversed this trend (Figure 6(g), arrow shown). These data demonstrated that HG induced mitochondrial malfunction and aggravated nuclear fragmentation of early apoptotic cells by activating the TLR4/NF-*κ*B signaling pathway.

### 2.7. Overexpression of PGC-1*α* Diminished HG-Induced Caspase-3 Expression in HK-2 Cells under the Inhibition of the TLR4/NF-*κ*B Signaling Pathway

**Figure 7:**
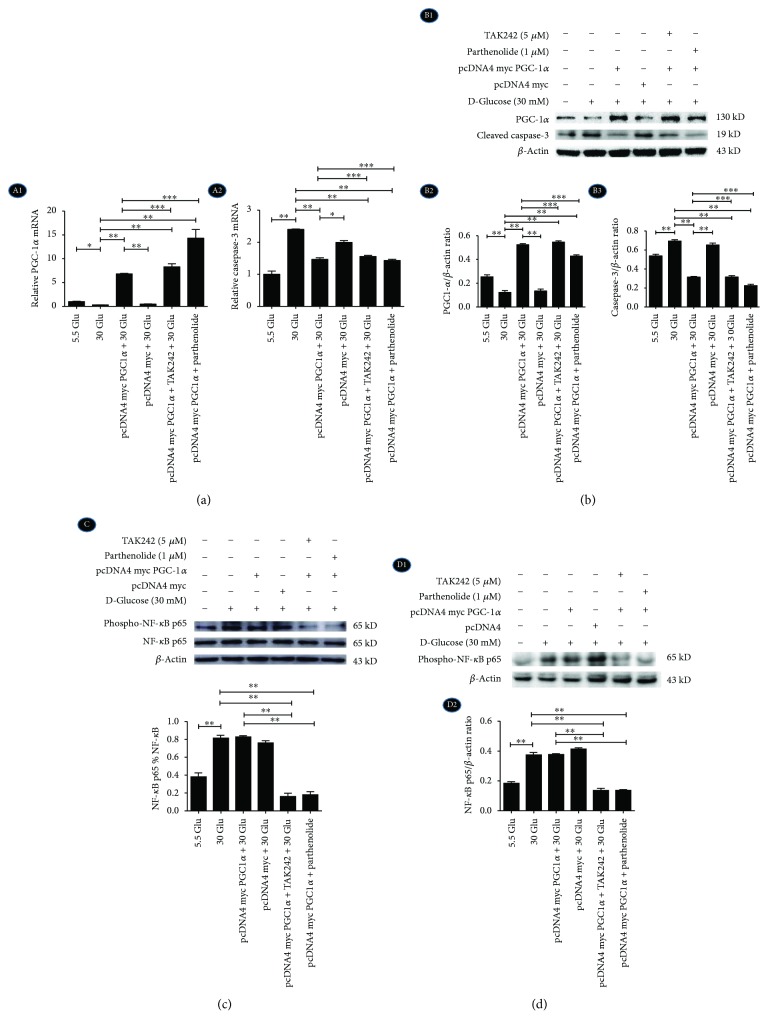
Overexpression of PGC-1*α* diminished HG-induced caspase-3 expression in HK-2 cells. HK-2 cells were transfected with pcDNA4 myc PGC-1*α* or pcDNA4 myc (empty vector) for 24 h. (a) A1: The mRNA level of PGC-1*α*. A2: The mRNA level of cleaved caspase-3. (b) B1-B2: The protein expression of PGC-1*α* and cleaved caspase-3 were examined by Western blotting. (c) The protein expression of NF-*κ*B p65 and phospho-NF-*κ*B p65 with HG for 2 h. (d) D1-D2: The protein expression of phospho-NF-*κ*B p65 with HG for 24 h. Each assay was representative of three independent experiments. Data were expressed as means ± SEM; ^∗^*P* < 0.05, ^∗∗^*P* < 0.01, and ^∗∗∗^*P* > 0.05.

HK-2 cells were divided into six groups with different treatments: (1) control group with 5.5 mM glucose (5.5 Glu), (2) high glucose group with 30 mM glucose (30 Glu), (3) PGC-1*α* plasmid group with 30 mM glucose (PGC-1*α* + 30 Glu), (4) PGC-1*α* empty plasmid group with 30 mM glucose (empty vector + 30 Glu), (5) PGC-1*α* plasmid with 30 mM glucose and TAK242 (PGC-1*α* + TAK242 + 30 Glu), and (6) PGC-1*α* plasmid with 30 mM glucose and parthenolide (PGC-1*α* + parthenolide + 30 Glu). HG increased the expression of cleaved caspase-3, but PGC-1*α* overexpression reduced the level of cleaved caspase-3 in mRNA (Figure 7(a), A2) and protein levels (Figure 7(b), B1–B3), supporting the notion that PGC-1*α* overexpression is involved in HG-induced cell apoptosis. Furthermore, the results also demonstrated that phospho-NF-*κ*B p65 increased in HK-2 cells with 30 mM HG for 2 h (Figure 7(c)) and 24 h (Figure 7(d)), while it did not decrease in PGC-1*α*-overexpressed cells, which further proved that PGC-1*α* might be the downstream protein of the TLR4/NF-*κ*B signaling pathway in HG-induced changes in HK-2 cells.

### 2.8. Overexpression of PGC-1*α* Restored HG-Induced Mitochondrial Membrane Potential (△*Ψ*_m_) Alteration and Apoptosis in HK-2 Cells under the Inhibition of the TLR4/NF-*κ*B Signaling Pathway

**Figure 8:**
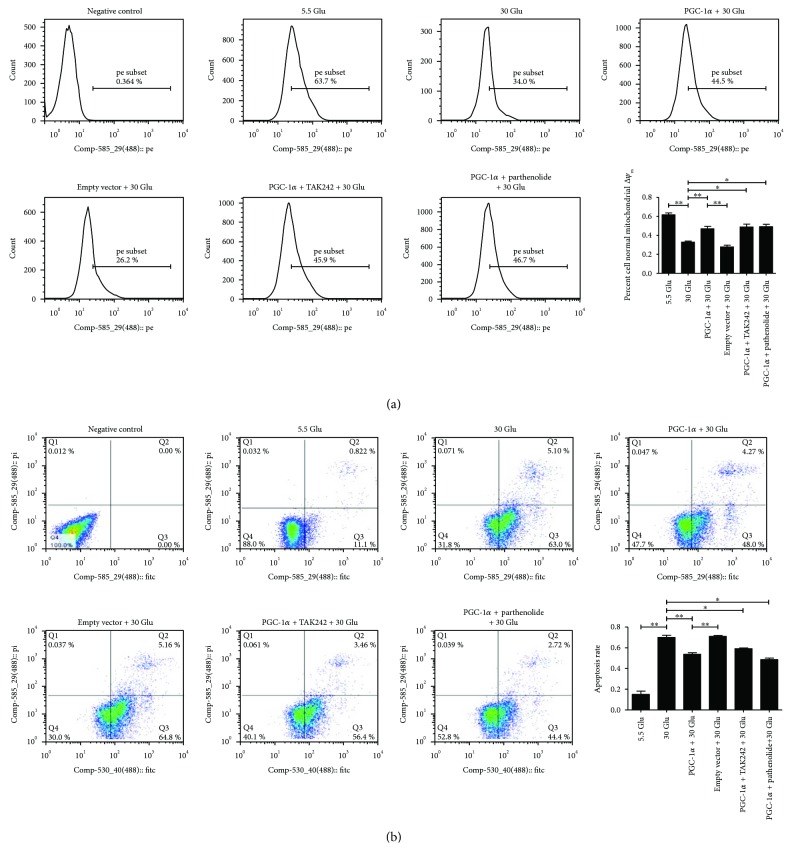
Overexpression of PGC-1*α* restored HG-induced mitochondrial △Ψ_m_ alteration and apoptosis. HK-2 cells were transfected with pcDNA4 myc PGC-1*α* for 24 h; the PGC-1*α*-overexpressed cells were treated with TAK242 for 2 h prior to HG (30 mM) treatment, with parthenolide and HG (30 mM) or with HG (30 mM) for 24 h. Mitochondrial △Ψ_m_ (a) and apoptosis (b) of HK-2 cells were analyzed by flow cytometry analysis. Each assay was representative of three independent experiments. Data were expressed as means ± SEM; ^∗^*P* < 0.05 and ^∗∗^*P* < 0.01.

By flow cytometry analysis, a loss of mitochondrial △Ψ_m_ detected by TMRM staining was observed under 30 mM HG ambience in cells undergoing early apoptosis, which was recovered to baseline with the overexpression of PGC-1*α* (Figures 8(a), A). In addition, the apoptotic rate was only 11.92% with 5 mM D-glucose in HK-2 cells, but peaked at 68.1% in the 30 Glu group (Figures 8(b), B), which was agreed with the reported literature that high glucose led to increased cell apoptosis. However, the apoptotic rate was, respectively, 52.27% in the “PGC-1*α* + 30 Glu group,” 59.86% in the “PGC-1*α* + TAK242 + 30 Glu group,” and 47.12% in the “PGC-1*α* + parthenolide + 30 Glu group,” which were significantly decreased when compared with that of the 30 Glu group. Statistics indicated that PGC-1*α* overexpression inhibited HG-induced loss of mitochondrial △Ψ_m_ and apoptosis in HK-2 cells.

## 3. Discussion

Recently, researches have demonstrated that mitochondria-related oxidative damage and apoptosis play a key role among the multifactorial pathogenesis of DKD patients [13, 14]. In addition, TLR4 has been previously described involving in hyperglycemia-induced inflammatory state of renal tubulus in vitro and vivo [15, 16], but its role in oxidative damage and apoptosis in renal tubular cells in DKD remains unclear. This study describes a cascade event that links TLR4/NF-*κ*B activation to mitochondria-related oxidative damage and apoptosis through downregulation of PGC-1*α* in renal tubular cells under HG condition.

There are increasing evidences showing the significance of the TLR4 pathway in the development of DKD [17]. However, most of them were focusing on its effect related to the tubulointerstitial inflammatory response [4, 12]. In this study, we found that TLR4 was extensively expressed in tubular cells in the kidney of patients with DKD and was coexistent with fragmented mitochondria. Further analysis revealed a positive correlation between TLR4 expression and tubular injury. These results indicated that TLR4 might play an important role in mitochondria-related tubular oxidative damage in DKD, besides its activation effect of inflammatory response. This speculation was further identified by a diabetic dbdb mouse model in our study.

Many studies have reported TLR4 to be critical for the activation of NF-*κ*B and subsequent production of proinflammatory cytokines implicated in diseases [18, 19]. Molecular silencing of TLR4 in tubular cells with siRNA attenuated HG-induced I*κ*B/NF-*κ*B activation, indicating that the TLR4-NF-*κ*B signal pathway plays an important role in diabetic nephropathy [20, 21]. In this study, we have also proved that the TLR4 inhibitor could effectively decrease the expression of phospho-NF-*κ*B p65 increased in HK-2 cells under HG conditions, indicating that NF-*κ*B is the downstream signal molecule of TLR4 in tubular cells.

Mitochondrial dysfunction has proved to be a contributing factor in the pathogenesis of DKD [22]. Mitochondria-related oxidative damage could result in the release of mitochondrial cytochrome c and activation of caspase-3, leading ultimately to apoptosis [23]. In order to investigate the mechanism for the effect of TLR4/NF-*κ*B on the mitochondria-related tubular oxidative damage in hyperglycemia, we inhibited the expression of TLR4 or NF-*κ*B in vivo and in vitro. We observed a partial rescue of HG-induced deformation of mitochondria, inhibition of ATP production, depressed mitochondrial respiration and activity of antioxidant enzymes, and increased caspase-3 and cytoplasm cytochrome c, which indicated that inhibition of the TLR4/NF-*κ*B signaling pathway could protect mitochondrial dysfunction in the tubular cells of diabetic rats and in cultured tubular cells induced by HG. These beneficial effects on tubular cells reverse ROS generation and apoptosis and protect tubular cells from oxidative injury by HG in vivo and in vitro.

Researches have identified mitochondrial fragmentation as a novel mechanism contributing to mitochondrial damage and apoptosis in vivo in mouse models of disease [24]. In tubular cells, a significant portion of mitochondria line up perpendicular to the basement membrane, making an excellent model for studying mitochondrial fragmentation in vivo. Cross sections of tubules normally show 10%–20% longitudinally sectioned long mitochondria, while they appear as short rods or spherical fragments in pathological apoptosis in disease models. Fragmented mitochondria were found to re-fuse if the injurious stress is removed before permanent injury happens to trigger apoptosis [25]. In this study, we observed that most of the mitochondria within the injured tubules in renal biopsy tissues and diabetic mice were fragmented and had a randomly disorganized cellular distribution, which could be reversed by inhibition of the TLR4/NF-*κ*B inhibitor, indicating that the TLR4/NF-*κ*B signaling pathway induced mitochondrial fragmentation, which contributes to subsequent tubular cell apoptosis.

As a main regulator of mitochondrial function, PGC-1*α* exerts a rescuing effect in substance metabolism and oxidative metabolism by regulating mitochondrial biogenesis and ROS scavenging enzymes [26]. An increased PGC-1*α* expression may protect cells from oxidative stress and oxidative stress-mediated cell apoptosis [27, 28]. In cardiac myocytes, Schilling et al. showed that TLR4 activation could lead to the phosphorylation and nuclear translocation of NF-*κ*B, triggering cardiac energy metabolic reprogramming by repressing genes encoding PGC-1*α*, and the absence of TLR4 abolished LPS-induced downregulation of PGC-1*α*, indicating that the suppression of PGC-1*α* was shown to occur through a TLR4- and NF-*κ*B-dependent mechanism [29]. In this study, we observed that PGC-1*α* was decreased in a diabetic mouse model and was reversed after the inhibition of TLR4 *in vivo*. In HK-2 cells, overexpression of PGC-1*α* inhibited a series of HG-induced changes including downregulation of mitochondrial membrane potential, upregulation of apoptosis-related protein cleaved caspase-3, and cell apoptosis directly, verifying the protective function of PGC-1*α* to mitochondrial function and cell survival in HG condition. Moreover, PGC-1*α* overexpression did not decrease HG-induced TLR4 activation and NF-*κ*B p65 phosphorylation, further confirming that PGC-1*α* is a downstream protein of TLR4/NF-*κ*B, which protected renal tubular cells from HG-induced mitochondria-related oxidation and apoptosis.

In conclusion, TLR4 plays a significant role in HG-induced mitochondrial dysfunction, mitochondria-related oxidation, and apoptosis by regulating downstream protein PGC-1*α* in RTEC, hoping to provide a better understanding and a more effective therapeutic approach for the prevention and treatment of DKD.

## 4. Materials and Methods

### 4.1. Main Reagents and Materials

Human kidney proximal tubular epithelial cells (HK-2) were a cell line purchased from the American Type Culture Collection (ATCC, USA). Antibodies were from the following sources: polyclonal anti-TLR4 from Abcam (USA). Monoclonal anti-PGC-1*α*, monoclonal anti-caspase-3, and NF-*κ*B p65 were from Cell Signaling Technology (Boston, USA). Polyclonal phospho-NF-*κ*B p65 (ser536) antibody was from Bioworld (USA), and polyclonal anti-cytochrome C was from Proteintech (Wuhan, China). Beta-actin and all secondary antibodies for Western blot and immunofluorescence were from Proteintech (Wuhan, China). TAK242 was from MedChem Express (USA), and parthenolide was from Sigma (USA). Plasmids containing pcDNA4 myc PGC-1*α* (pcDNA4/PGC-1*α*) was bought from Addgene (USA). Lipofectamine 2000, MitoTracker Red CMXRos, MitoSOX, and TRIzol were purchased from Invitrogen (USA). PrimeScript™ RT reagent Kit with gDNA Eraser and SYBR® Premix Ex Taq™ (Tli RNase H Plus) were from TaKaRa (Japan). Annexin V-FITC Apoptosis Detection Kit was from Beyotime (Shanghai, China). TMRM Detection Kit was from Genmed Scientifics Inc. (USA). Other reagents, including DMEM/F12 medium, bovine serum albumin (FBS), and trypsin, were obtained from Gibco (USA).

### 4.2. Morphological Analysis of Kidney

12 patients with DKD and 12 non-DKD controls (normal kidney tissue) were recruited for this study. Human renal biopsy tissues from the 24 cases were studied by staining of PAS and PASM. A semiquantitative scoring system was used to evaluate the tubulointerstitial lesion index, and tubular damage was also scored [30, 31]. All procedures were carried out in accordance with the approved guidelines. All patients did not use adrenal cortical hormones or immunosuppression. The institutional review board and the administrators of the Department of Nephrology in The Second Xiangya Hospital approved the protocol for this study. An informed consent was obtained from all the participants.

The kidney tissue of mice was routinely processed, embedded in paraffin, and sectioned at 2–3 mm thickness, deparaffinized, and rehydrated using standard techniques. Mouse sections were stained with hematoxylin-eosin stain (HE).

### 4.3. Examination of Mitochondrial Fragmentation in Renal Tissue and HK-2 Cells

The alterations of mitochondria in renal tubules were gauged by electron microscopy (EM). Mitochondria having a length < 1 *μ*m and spherical configuration were identified as fragmented. We determined the percentage of cells that had less than 1% long filamentous mitochondria to indicate the degree of mitochondrial fragmentation in patients and mice [24].

MitoTracker Red CMXRos was used for the evaluation of mitochondrial morphology in HK-2 cells. The mitochondria within a cell were often either filamentous or fragmented. In cases of mixed mitochondrial morphology, we classified the cells based on the majority (>70%) of mitochondria, according to earlier studies. For each sample, several random fields of cells (≥100 cells per dish) were evaluated [32, 33].

### 4.4. Animal Experimental Design

A total of 10 male dbm mice and 20 adult male dbdb mice at 16 weeks of age (body weight 32–40 g) were divided into three groups of 10 animals each. The first group was male dbm mice, which served as a control. The second group of dbdb mice received an intraperitoneal injection with vehicle alone (dbdb group). The third group included dbdb mice which received an intraperitoneal injection of TLR4 inhibitor TAK242 (3 mg/kg for 7 days). All animals were killed at 17 weeks following administration. The Institutional Animal Experimentation Ethics Committee as described above approved the animal experimental protocols.

### 4.5. Cell Culture

Human kidney proximal tubular epithelial cells (HK-2), an immortalized cell line from the American Type Culture Collection (ATCC, USA), were used in this study. HK-2 cells were cultured in DMEM/F12 medium supplemented with 10% FBS, penicillin 1 × 10 5 U/L, and streptomycin 100 mg/L. Until being seeded at 80–90% confluence, the cells were exposed to different concentrations of D-glucose and TLR4/NF-*κ*B blockers, and concrete interventions and groupings were as follows: A: 5.5 mM D-glucose (control/5.5 Glu group), B: 30 mM D-glucose (30 Glu group), C: TLR4 inhibitor (TAK242) and 30 mM D-glucose (TAK242 + 30 Glu group), and D: NF-*κ*B blocker (parthenolide) and 30 mM D-glucose (parthenolide + 30 Glu group).

### 4.6. Immunohistochemistry (IHC)

Renal tissue sections from human and mice for immunostaining were deparaffinized and rehydrated. Immunohistochemistry was performed using anti-TLR4 antibody (1 : 100, Abcam, USA), caspase-3 (1 : 100, CST, Boston, USA), and cytochrome c (1 : 100, CST, Boston, USA) antibody as a primary antibody followed by a secondary antibody. Then, slides were visualized by using a DAB detection kit according to the manufacturer's instructions, and the tissue specimens were examined by light microscopy.

For human and mouse tissue sections, the average intensity from at least 20 randomly selected fields was measured using ImageJ software (National Institutes of Health, Bethesda, MD).

### 4.7. ATP Assay

ATP levels were determined in cell lysates obtained from mouse renal tissue and HK-2 cells using an ATP Assay Kit (Genmed, Shanghai, China). The assays were run according to the manufacturer's instructions. The values were expressed as fold change compared to the control.

### 4.8. Respiratory Chain Complex III Activity

Mitochondrial respiratory chain complex III activity was measured by using a spectrophotometer (Genmed, Shanghai, China) according to the manufacturer's instruction. The values were expressed as fold change compared to vehicle control.

### 4.9. Mitochondrial Enzyme Activities

The Superoxide Dismutase Activity Assay Kit (Alexis Biochemicals) and Catalase Activity Colorimetric/Fluorometric Assay Kit (BioVision Inc.) were used for the detection of Mn-SOD and catalase activity, following the manufacturer's guidelines. Enzyme activities were displayed as fold change compared to the control.

### 4.10. Measurements of Superoxide Generation and Apoptosis

DHE was used for the detection of mitochondrial superoxide generation in vivo. A specific mitochondrial superoxide indicator MitoSOX red (Molecular Probes) was used for the detection of ROS production in HK-2 cells in vitro. 20 randomly selected fields were photographed for tissue sections, and the mean fluorescence intensity was calculated by using NIH ImageJ software and was expressed relative to the control (set as 1).

The TUNEL procedure and Hoechst 33258 staining were used to detect apoptosis following the manufacturer's instructions. 10 random fields of cells (approximately 100 cells per group) were counted to determine the percentage of cells undergoing apoptosis.

### 4.11. PGC-1*α* Overexpression

To enforce PGC-1*α* expression in HK-2 cells, pcDNA4 myc PGC-1*α* was bought from Addgene (USA). Then, pcDNA4 myc PGC-1*α* was purified using Plasmid Kit (Qiagen, USA). HK-2 cells were seeded at 70% confluence, and 2.5 *μ*g of pcDNA4 myc PGC-1*α* or pcDNA4 myc was introduced into HK-2 cells using Lipofectamine 2000 (Invitrogen, USA) on 6-well culture dishes according to the manufacturer's instructions. After 24 h cultivation, the cells were exposed to different concentrations of D-glucose and TLR4/NF-*κ*B blockers, and concrete interventions and groupings were as follows: a: 5.5 mM D-glucose (control/5.5 Glu group); b: 30 mM D-glucose (30 Glu group); c: pcDNA4 myc PGC-1*α* and 30 mM D-glucose (PGC-1*α* + 30 Glu group); d: pcDNA4 myc and 30 mM D-glucose (empty vector + 30 Glu group); e: pcDNA4 myc PGC-1*α*, TAK242, and 30 mM D-glucose (PGC-1*α* + TAK242 + 30 Glu group); and f: pcDNA4 myc PGC-1*α*, parthenolide, and 30 mM D-glucose (PGC-1*α* + parthenolide + 30 Glu group).

### 4.12. Real-Time Reverse Transcription Polymerase Chain Reaction (Real-Time RT-PCR)

Total RNA was isolated with TRIzol (Invitrogen, USA), and 1 *μ*g RNA was used for reverse transcription to generate template cDNA. The relative mRNA levels were determined via fluorogenic quantitative PCR, and *β*-actin served as an internal reference gene. Specific primers for the use of SYBR Green are as follows: TLR4: 5′-ACCTGTCCCTGAACCCTATG-3′ (forward) and 5′-TCTAAACCAGCCAGACCTTGA-3′ (reverse); NF-*κ*B: 5′-AGCACAGATACCACCAAGACC-3′ (forward) and 5′-CGGCAGTCCTTTCCTACAAG-3′ (reverse); PGC-1*α*: 5′-TGAGTCTGTATGGAGTGACATCG-3′ (forward) and 5′-ACTTGAGTCCACCCAGAAAGC-3′ (reverse); and caspase-3: 5′-TGCATACTCCACAGCACCTG-3′ (forward) and 5′-TTCTGTTGCCACCTTTCGGT-3′ (reverse). The primer sequences were designed using Primer 5.0 and were searched for specificity. Real-time quantitation was performed on the Applied Biosystems® 7300 system (ABI 7300, USA). The PCR parameters were as follows: 95°C for 30 s followed by 40 cycles of denaturation at 95°C for 5 s and annealing at 60°C for 31 s. The quantitative PCR results were calculated using the 2^−ΔΔCt^ methods.

### 4.13. Western Blotting (WB)

The frozen kidney tissues of mice were lysed with RIPA lysis buffer (Beyotime, Shanghai, China) followed by centrifugation at 12000 rpm at 4°C to obtain cellular proteins in the supernatant. Cell lysate of HK-2 was obtained using RIPA buffer (Beyotime, Shanghai, China) and cocktail (Roche Diagnostics, Mannheim, Germany). The extractions of cell cytoplasmic and mitochondrial fractions were obtained by Mitochondria/Cytosol Fractionation Kit (Abcam, USA) according to the manufacturer's protocol. The protein concentration was quantified using the BCA method (Beyotime, Shanghai, China). Then, the samples were separated in 10% SDS-polyacrylamide gels, transferred onto polyvinylidene difluoride membranes (Millipore, MA, USA), and incubated overnight at 4°C with primary antibodies. After overnight incubation, membranes were washed 3 times after which they were incubated with secondary antibodies (anti-mouse IgG, Proteintech, Wuhan, China; anti-rabbit IgG, Proteintech, Wuhan, China) for 1 h at room temperature and again washed 3 times. The blots were then detected using ECL (Millipore, MA, USA). Primary antibodies used in this experiment were anti-TLR4 antibody (1 : 1000, Abcam, USA), NF-*κ*B p65 antibody (1 : 1000, CST, Boston, USA), phospho-NF-*κ*B p65 (ser536) antibody (1 : 1000, Bioworld, USA), cytochrome C (1 : 1000, CST, Boston, USA), monoclonal anti-PGC-1*α* (1 : 1000, CST, Boston, USA), and anti-caspase-3 (1 : 1000, CST, Boston, USA). The intensity of each band was estimated using NIH image software and was normalized to *β*-actin.

### 4.14. Assessment of Mitochondrial Membrane Potential (△Ψ_m_)

Mitochondrial △Ψ_m_ of cells was assessed by TMRM Detection Kit (Genmed Scientifics Inc., USA). HK-2 cells plated on 24-well culture dishes were harvested after trypsinization when they were seeded at 70% confluence, and then the cells were stained with TMRM dyeing liquid according to the manufacturer's instruction. For 20 min incubation in the dark at 37°C, mitochondrial △Ψ_m_ of cells was examined by a FACSCalibur flow cytometer (BD Biosciences, San Jose, USA). The fluorescence intensity of TMRM was monitored at 575 nm.

### 4.15. Flow Cytometry Analysis of Apoptosis

Cell apoptosis was measured with Annexin V-FITC Apoptosis Detection Kit (Beyotime, Shanghai, China). According to the manufacturer's instructions, cells were incubated with 195 *μ*L binding buffer containing 5 *μ*L Annexin V-FITC and 10 *μ*L propidium iodide in the dark for 20 min at room temperature. Cell apoptosis was analyzed on a FACSCalibur flow cytometer (BD Biosciences, San Jose, USA).

### 4.16. Data Analysis

Statistical analysis was carried out using the SPSS 20 software. Results were expressed as mean values ± standard error of the mean (SEM). Statistical differences among groups were analyzed by one-way ANOVA, and two-tailed *P* values are reported. *P* values less than 0.05 were considered statistically significant.

## Figures and Tables

**Table 1:** Clinical characteristics of the patients.

	N-DKD	DKD
Sex (male/female)	4/8	5/7
Age (year)	24.92 ± 3.50	45.42 ± 4.01^∗^
Blood glucose (mmol/L)	4.60 ± 0.25	8.69 ± 1.03^∗^
Urine protein (g/24 h)	1.71 ± 0.99	5.87 ± 1.02^∗^
Serum creatinine (*μ*mol/l)	66.74 ± 9.67	174.48 ± 27.68^∗^
Triglyceride (mmol/L)	1.40 ± 0.38	2.06 ± 0.31

Values are means ± SE; ^∗^*P* < 0.05, compared with N-DKD.

**Table 2:** Physical and metabolic parameters in mice.

	db/m	db/db	db/db + TAK242
Body weight (g)	35.9 ± 0.52	64.00 ± 5.02^∗^	58.42 ± 1.54^∗^
Blood urea nitrogen (mmol/L)	5.59 ± 0.36	11.42 ± 0.88^∗^	7.04 ± 0.38^∗∗^
Serum creatinine (*μ*mol/L)	3.76 ± 0.19	16.77 ± 0.92^∗^	8.91 ± 0.35^∗∗^
Urine protein (mg/24 h)	7.53 ± 0.35	26.30 ± 1.15^∗^	15.31 ± 0.66^∗∗^
ACR (*μ*g/mg Cr)	0.23 ± 0.01	2.89 ± 0.07^∗^	2.76 ± 0.08^∗^

Values are means ± SE; ^∗^*P* < 0.05, compared with the db/m group; ^∗∗^*P* < 0.05, compared with the db/db group; urinary albumin : creatinine ratio (ACR).
